# Genome sequences of *G. rubripertincta* bacteriophages Eddiemania and UBSmoodge isolated in Las Vegas, Nevada

**DOI:** 10.1128/mra.00019-25

**Published:** 2025-03-05

**Authors:** Maren M. Mandel, Victoria L. Dudek, Ethan D. Handelman, Ryal Tilleth, Kendra W. Kimberley, Chelsey C. McKenna, James R. Theoret, Earl J. Yoon, Erin J. Windsor

**Affiliations:** 1 Department of Biological Sciences, College of Southern Nevada 170249 , Las Vegas, Nevada, USA; DOE Joint Genome Institute, Berkeley, California, USA

**Keywords:** bacteriophage genetics, *Gordonia*, genome analysis

## Abstract

Eddiemania and UBSmoodge are bacteriophages isolated on *Gordonia rubripertincta* NRRL B-16540. Eddiemania has a 3′ sticky overhang, a 61,427 bp genome predicted to encode 92 putative genes, and a siphovirus virion morphology. The UBSmoodge genome was identified as a circularly permuted, 92,786 bp long genome predicted to encode 130 putative genes and having a myovirus virion morphology.

## ANNOUNCEMENT

Bacteriophages Eddiemania and UBSmoodge were isolated from soil and characterized by the College of Southern Nevada through the Science Education Alliance-Phage Hunters Advancing Genomics and Evolutionary Science (SEA-PHAGES) program (1). The host bacterium, *Gordonia rubripertincta*, has potential uses in bioremediation, grows well in the laboratory at 30°C, and is stored long term at −20°C (2). Eddiemania was collected at a park in Las Vegas and came from direct isolation. UBSmoodge was collected in a neighborhood in Las Vegas and came from an enriched sample. Protocols for the isolation, purification, and DNA extraction of bacteriophages came from the SEA-PHAGES *Phages Discovery Manual* (3). Soil samples were incubated in PYCa media with amphotericin B at 30°C and shaken at 250 rpm for 1 hour. The supernatant was filtered through a 0.22 µM filter. For direct isolation, the supernatant was used directly for plaque assays. For enriched isolation, the 10 mL of supernatant was added to 500 µL of host bacterium and incubated at 30°C for 3 days while on the rocker at 220 rpm. The sample was filtered through a 0.22 µm filter prior to the plaque assay. After completing three rounds of purification and one round of amplification, DNA was extracted from high-titer lysates using the Norgen phage DNA isolation kit with five rounds of freeze/thaw (4 minute freeze in a dry-ice ethanol bath and a 1 minute thaw). Bacteriophages were sequenced at the Pittsburgh Bacteriophage Institute using an Illumina MiSeq instrument. Sequencing libraries were created from extracted genomic DNA using the New England Biolabs Ultra II Library kit v.3, 150-base single-end reads according to the manufacturer’s instructions. Raw reads were uploaded to Newbler v.2.9 (454 Life Sciences, Roche) using default settings to produce continuous reads (4). Reads were analyzed with Consed v.2.9 using default settings to produce a single contig, which was evaluated for accuracy and completeness by looking for gaps and low-consensus areas (http://bozeman.mbt.washington.edu/consed/consed.html (4). Bacteriophage genomic termini were identified using the Pileup Analysis Using Starts and Ends workflow in Galaxy (https://cpt.tamu.edu/) (4). Concentrated purified lysate was spotted on a copper grid, stained with Uranyless acetate, and sent to Northern Arizona University for electron microscope imaging (3).

Annotations were completed using the following programs: DNA Master v.2.53.6 (http://cobamide2.bio.pitt.edu/computer.htm), Starterator v.1.2 (https://github.com/SEA-PHAGES/starterator), Phamerator (https://phamerator.org/) (5), PhagesDB BLAST (https://phagesdb.org/blast/) (1), National Center for Biotechnology Information (NCBI) BLAST (databases: BLASTtn Standard Core Nucleotide and BLASTp Standard Non-redundant) (6), PECAAN (7), GeneMark v.2.5p (8), Glimmer v.3.02 (9), Aragorn v.1.1 and v.1.2.38 (10), HHPRED (databases: PDB, NCBI-CD, PFam-A, and SCOPe) (11), tRNAscan-SE v.2.0 (12), and DeepTMHMM v.1.0.24 (13) using the default parameters found in the SEA-PHAGES bioinformatics guide (14).

**Fig 1:**
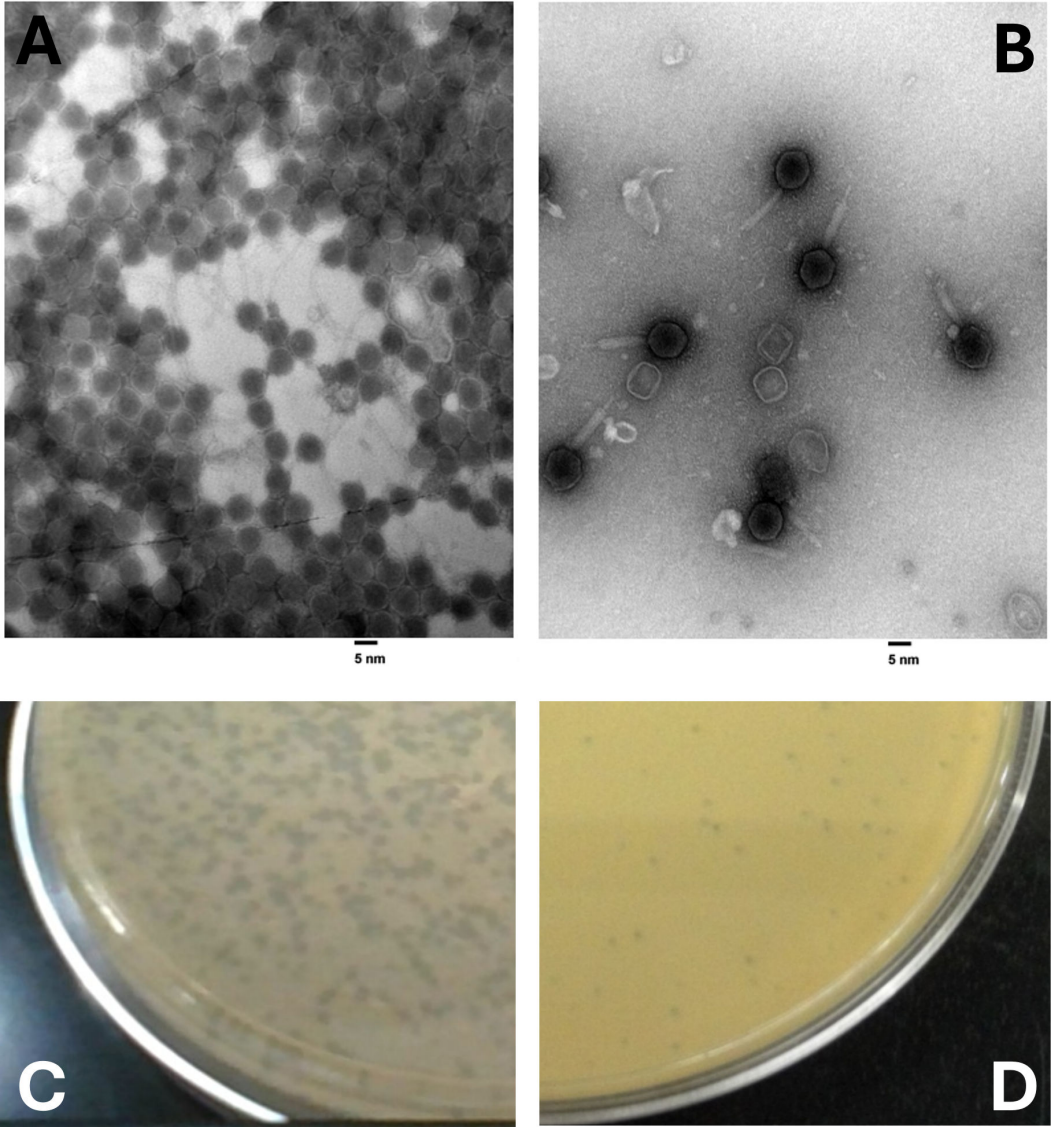
Transmission electron micrographs of bacteriophages Eddiemania (**A**) and UBSmoodge (**B**) reveal morphologies characteristic of the siphovirus and myovirus morphology, respectively. Negative staining with Uranyless acetate. Magnification: ×40,000. (C) Plaques of Eddiemania measuring 1–2 mm in diameter. (D) Plaques of UBSmoodge measuring 0.5–1.0 mm in diameter.

Eddiemania, with siphovirus morphology (Fig. 1A), had 92 genes identified, 24 of which were assigned putative functions. UBSmoodge, with myovirus morphology (Fig. 1B), had 130 genes, 36 of which had putative functions assigned. UBSmoodge has four genes in its reverse reading frame and a gap of 1,178 bp when switching from its reverse reading frame to its forward reading frame. These four reverse genes and a 1000 + bp gap are characteristic of the most closely related bacteriophages (Table 1).

**TABLE 1:** Actinobacteriophage genome and sequencing information

Phage	GenBank accession number	SRA^*a*^ accession number	GPS coordinates	Isolation	Cluster	Genome length	GC content (%)	Genome end type	Sequencing coverage	Number of reads	Putative genes	Related phages
Eddiemania	PQ184802	SRR29377726	36.11021 N, 115.2384 W	Direct	DJ	61,427	51.6	3′ Sticky overhang -CGCCGCTCT	723	313,409	92	Burly DJ (ON526971) Hydrus DJ (OQ938589)
UBSmoodge	PQ201087	SRR29377732	36.233 N, 115.083 W	Enriched	DQ	92,786	60.2	Circularly permuted	167	109,568	130	FlyingTortilla DQ (PQ184803) Scarlet Raider DQ (PQ184837)

^*a*^ SRA, Sequence Read Archive.

## Data Availability

Eddiemania GenBank and Sequence Read Archive (SRA) accession numbers are PQ184802 and SRR29377726, respectively. UBSmoodge GenBank and SRA accession numbers are PQ201087 and SRR29377732, respectively.
